# The relationship between cerebral blood flow and venous sinus pressure: can hyperemia induce idiopathic intracranial hypertension?

**DOI:** 10.1186/s12987-021-00239-2

**Published:** 2021-02-04

**Authors:** Alexander Robert Bateman, Grant Alexander Bateman, Tracie Barber

**Affiliations:** 1 School of Mechanical Engineering, University of New South Wales, Library Rd, Kensington, NSW 2052 Australia; 2 Department of Medical Imaging, John Hunter Hospital, Newcastle, NSW Australia; 3 Newcastle University Faculty of Health, Callaghan Campus, Newcastle, NSW Australia

**Keywords:** Computational fluid dynamics, Venous sinus, Cerebral blood flow, Idiopathic intracranial hypertension

## Abstract

**Background:**

It has been shown that idiopathic intracranial hypertension (IIH) in children is associated with cerebral hyperemia, which induces an increase in cerebral venous pressure. The current literature suggests venous pressure scales with blood flow in a linear fashion, however, a linear relationship would not raise the pressure high enough to induce IIH. There is, however, some evidence to suggest that this relationship could be quadratic in nature. The purpose of this paper is to characterize the relationship between cerebral blood flow and the pressure drop across the cerebral venous system.

**Methods:**

10 CT venogram data sets were collected for this study, with 5 useable geometries created. Computational fluid dynamics (CFD) models were generated using these geometries, with 10 simulations conducted per patient. The flow rates tested ranged from 200 mL/min to 2000 mL/min. 3D pressure and velocity streamline distributions were created and analyzed for each CFD model, with pressure drops across the cerebral venous system determined. The effective and hydraulic diameters were determined at the superior sagittal sinus, transverse sinus and both proximal and distal sigmoid sinuses.

**Results:**

A quadratic relationship between blood flow and sinus pressure was found, with correlations of 0.99 or above in all five patients. The presence of vortical blood flow was found to explain this trend, with fluid curl and pressure drop correlations being above 0.97. This suggests that the presence of high blood flow should be considered in the diagnostic workup of IIH.

**Conclusions:**

The cerebral venous sinus blood flow and pressure response relationship are quadratic in nature, with the major cause of this being the degree of rotation induced in the flow. The elevated blood flow found in children with IIH can explain the increased ICP that is found, secondary to the increase in venous pressure that develops.

## Background

Idiopathic intracranial hypertension (IIH) is defined as an increase in the cerebrospinal fluid (CSF) pressure above 18.34 mmHg in adults or children who are not obese or sedated, due to an unknown cause [1]. Diagnosis of IIH typically includes the fulfillment of criteria, which includes papilledema and, in some instances, venous sinus stenosis [2]. The presence of transverse venous sinus stenosis is typically evident in IIH patients and commonly indicates increased intracranial pressure (ICP) [2]. The relation is observed because the sinus stenosis elevates the superior sagittal sinus pressure, which is directly related to ICP [3]. This shows that factors which increase the pressure in the cerebral venous system, such as venous sinus stenosis, are important for consideration in the diagnosis of IIH. Intracranial pressure (ICP, units of mmHg) is defined by Davson’s equation:

1 $$ICP=FR_{CSF}\times R_{out}+SSS_{p}$$

where *FR*_*csf*_ is the CSF formation rate (mL/min), *R*_*out*_ is the CSF outflow resistance (mmHg.min/mL) and *SSS*_*p*_ is the superior sagittal sinus pressure (mmHg). Idiopathic intracranial hypertension (IIH) is characterised by an increase in the ICP, without the occurrence of parenchymal brain lesions, vascular malformations, hydrocephalus or CNS infection [4]. It has been shown that an increase in the CSF formation rate and CSF outflow resistance are not associated with IIH, but rather active hydrocephalus in children [5, 6]. Therefore, from Eq. 1, it can be observed that an increase in ICP in IIH must be attributed to an elevated venous pressure [7].

As fluids obey a law analogous to Ohm’s law, the superior sagittal sinus pressure is related to several variables, as shown in Eq. 2:

2 $${SSS}_{p}=TCBF\times {R}_{ven}+CVP$$

where *TCBF* is the total cerebral blood flow leaving the capillaries (mL/min), *R*_*ven*_ is the venous outflow resistance from the superior sagittal sinus to the jugular bulbs (mmHg.min/mL) and *CVP* is the central venous pressure (mmHg) [8]. An elevated central venous pressure has been shown to occur in IIH, due to obesity in adults [9] and the venous outflow resistance has been found to increase due to venous sinus stenosis, while the total cerebral blood flow is elevated in cerebral hyperemia [7]. In children suspected of IIH, the obesity rates do not differ significantly from controls and therefore, this is not a significant factor in IIH in children [10]. Cerebral hyperemia and mild venous sinus stenosis are, however, associated with the incidence of IIH [10], suggesting that cerebral hyperemia elevates the venous sinus pressure and ICP to a level whereby IIH could develop. The cause of cerebral hyperemia is not defined, although it is known that the blood flow is regulated to the metabolic demand of the neuronal tissue [11]. Thus, an increase in carbon dioxide or arterial blood pressure could increase the cerebral blood flow, neither of these appear to operate in this cohort [10]. The increase observed in the children with IIH would suggest a problem with blood flow regulation, but this would need further study.

Fall et al. estimated the venous outflow resistance by applying electrical circuit theory to the individual vessel resistance values [12]. This analysis assumed that the venous outflow resistance was constant, irrespective of the total cerebral blood flow. The model is therefore linear, as shown in Eq. 2, and an increase in TCBF will increase the superior sagittal sinus pressure by the multiple of the constant venous outflow resistance. This linear model, however, does not produce a venous pressure high enough to induce IIH in children, even with severe hyperemia. In a previous paper it was shown that in children of average age 8 years, the diagnosis of IIH requires the ICP to be increased by 3.8 mmHg above the baseline average ICP of 14.6 mmHg [10]. The average pressure drop across the venous system of 4.5 mmHg would need to be increased to 8.3 mmHg for IIH to be induced purely by hyperemia alone [10]. It was found that the cerebral blood flow in children at risk of IIH was 35 % higher than controls. Under a linear model, this would increase the baseline venous pressure drop of 4.5 mmHg by only 1.6 mmHg and would not be enough to induce IIH.

The relationship between ICP and TCBF may be non-linear, with a 33 % increase in ICP associated with a 17 % increase in TCBF [13]. By applying the three known values of the pressure drop across the venous system using the known arterial inflow from the literature for each, the resulting equation of best fit was found to be quadratic [10], suggesting that the venous pressure may be quadratically related to the TCBF and that the venous outflow resistance is not constant but increases with blood flow rate.

The purpose of this study is to determine if the relationship between TCBF and pressure response of a normal venous sinus system is quadratic, through the application of CFD. If this is shown to occur, then the fluid features of the cerebral venous system will be characterized, to discover the cause of this quadratic relationship. This may suggest that cerebral hyperemia could induce IIH in children.

## Methods

### Subjects

A total of 10 CT venogram data sets were collected for this study, with the patients selected from the International Stroke Perfusion Imaging Registry (INSPIRE). The registry database was searched to find all patients between 20 and 40 years of age, who had a contrast enhanced CT perfusion study whilst in the supine position, with the indication “rule out possible acute stroke”, in whom the subsequent study was shown to be entirely normal. There were 4 males and 6 females, of average age 30.4 ± 6.1. The perfusion protocol was acquired on a Toshiba Aquilion ONE and involved a 3D, 320 slice per-rotation acquisition with 0.5 mm through plane resolution and an in-plane resolution of 0.441 mm, resulting in a voxel size of 0.441 mm x 0.441 mm x 0.5 mm. Pre-contrast images were acquired, followed by a post intravenous contrast bolus data set obtained in the arterial, capillary and venous phases. In this study, the pre-contrast dataset was subtracted from the venous phase dataset using the scanners proprietary subtraction tool. The resulting 320 anonymized raw data slices were then exported for segmentation.

### Analysis

The geometric segmentation of the venous sinuses was performed using a semi-automatic process in ITK Snap [14]. This resulted in ten 3D patient geometries, which could be assessed for use in the study. Patients were excluded if there were discontinuous sections of the venous system due to resolution issues. Also, patients were excluded if the proximal internal jugular veins were not imaged, as the fluid flow through this section was required to adequately model the system. Finally, one patient was excluded due to the presence of a conjoining blood vessel between the sagittal and straight sinus. This is a finding which is not present in the majority of the population and therefore not a normal variant of the cerebral venous geometry [15]. Thus, five geometries were chosen as variations of a normal cerebral venous system. The five chosen geometries were refined to remove cortical blood vessels which were segmented during the original semi-automatic process.

The inlet of the superior sagittal and straight sinuses was positioned to where the known flow rates and pressures in the literature were measured [10]. The average sagittal sinus length was 10.78 ± 1.15 cm, with straight sinus length being 4.29 ± 0.23 cm. Smoothing of the geometry was performed using MeshLab (www.meshlab.net) to create a continuous system, which more accurately models the venous fluid flow. These processes were performed with the consultation of a neuroradiologist (GAB) to ensure that an appropriate representation of the cerebral venous systems was achieved. Flow extensions at the inlet and outlet sections were created using Meshmixer (http://www.meshmixer.com/), to ensure a fully developed flow profile was achieved before the test segments.

A polyhedral computational mesh was created in ANSYS Fluent Meshing (ANSYS, Canonsburg, Pennsylvania). The mesh sizes ranged between 2.6 and 3.4 million finite volumes. Steady flow CFD simulations were performed using ANSYS Fluent. A mass flow boundary condition was applied to the inflow, with flow rates ranging from 200 mL/min to 2000 mL/min, and a relative pressure outlet condition was prescribed. The straight sinus flow rate was set as being 30 % of the superior sagittal sinus as per the literature [8, 16]. The CFD simulations assumed Newtonian blood rheology and rigid sinus walls, with a fluid density of 1055$kg/{m}^{3}$ and viscosity of 0.0035 Pa.s. An appropriate turbulence model was applied to the simulations, ensuring that the blood flow features of the cerebral venous system were accurately resolved.

A verification procedure was performed to ensure that the finite sizes were sufficient, with residual thresholds also investigated to minimize computational errors. The flow patterns were compared to the literature, with the CFD simulations observed to effectively resemble physiological blood flow [17]. To test the accuracy of the models, the average pressure drop across the venous system of the five patients was compared to the known values from the literature, with associated errors below 3 % for all points [10]. This ensured that the CFD models effectively represented the physiologically normal cerebral venous system, in blood flow and pressure response.

3D pressure and velocity streamline distributions were created and analyzed for each CFD model. Values of pressure at the superior sagittal sinus, straight sinus and proximal internal jugular vein sections were calculated to determine the pressure drop over the cerebral venous system. The effective and hydraulic diameters were determined at the superior sagittal sinus, transverse sinus and both proximal and distal sigmoid sinus using a previously defined technique in the literature [10].

## Results

**Fig. 1:**
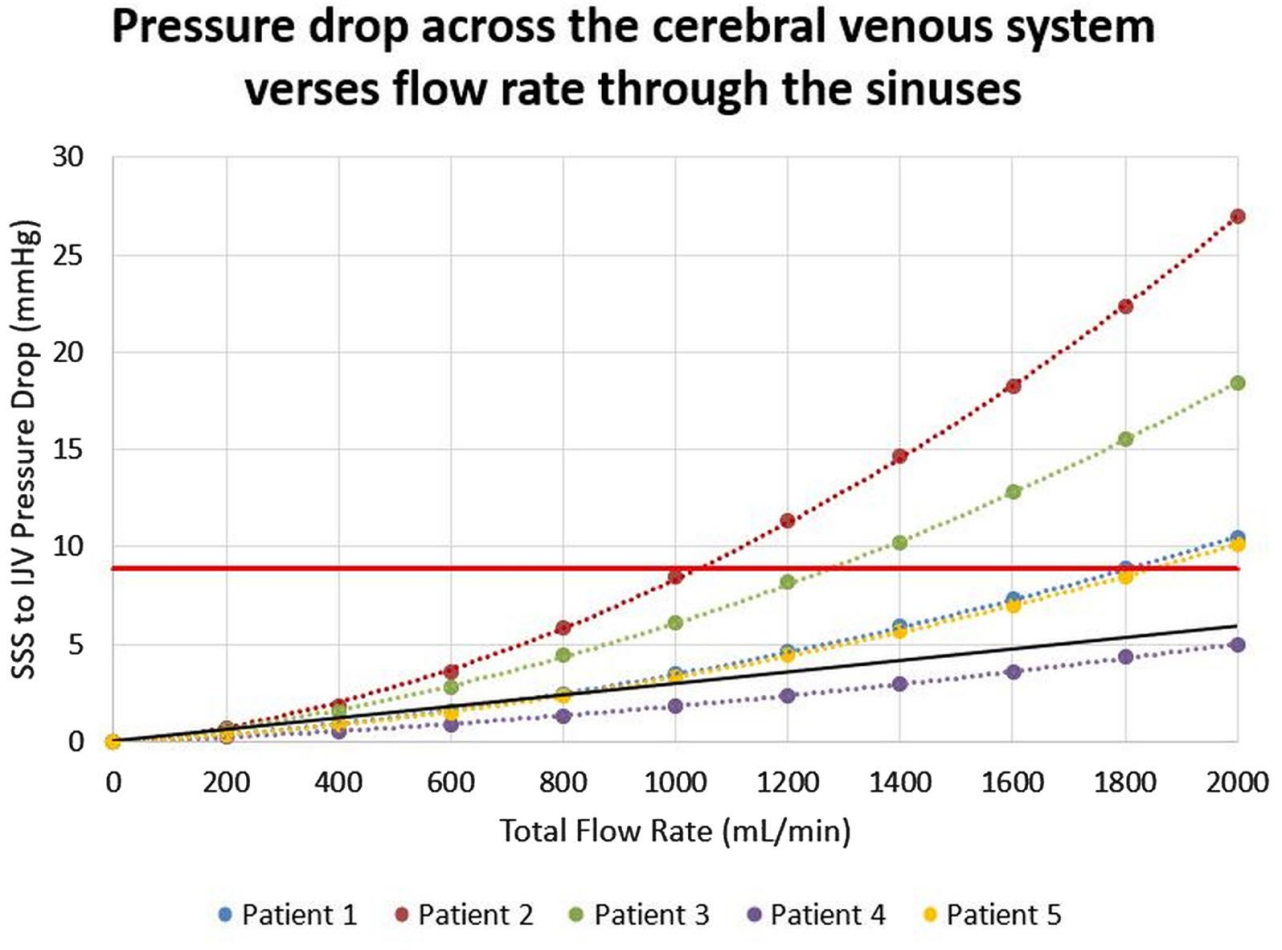
A graph of the pressure drop vs. flow rate relationship for the five patients. The dots represent the individual measurements. The red line is the threshold at which IIH would be induced i.e. 8.3 mmHg. The black line is the current linear model of flow rate vs. pressure. Note the black line never reaches the IIH threshold, however, 4 out of the 5 quadratic curves reach the threshold. *SSS* Superior Sagittal Sinus, *IJV* Internal Jugular Vein

**Fig. 2:**
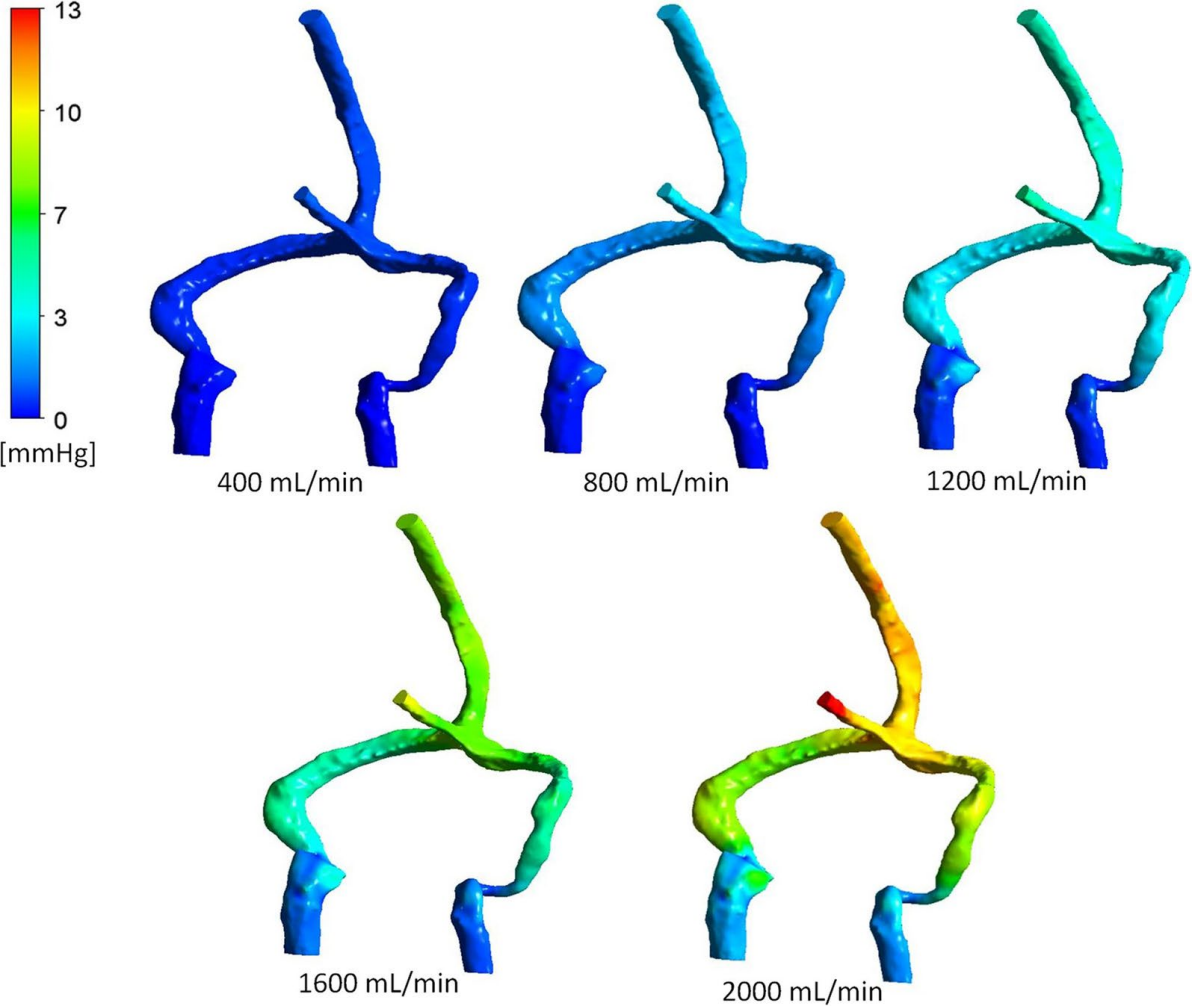
The CFD pressure distributions throughout the cerebral venous system of patient 1 for various flow rates. The pressures are colour coded to represent mmHg as per the scale. Note the pressure drop increases with blood flow, with the majority of the drop occurring across the distal sigmoid to jugular bulb segment

**Fig. 3:**
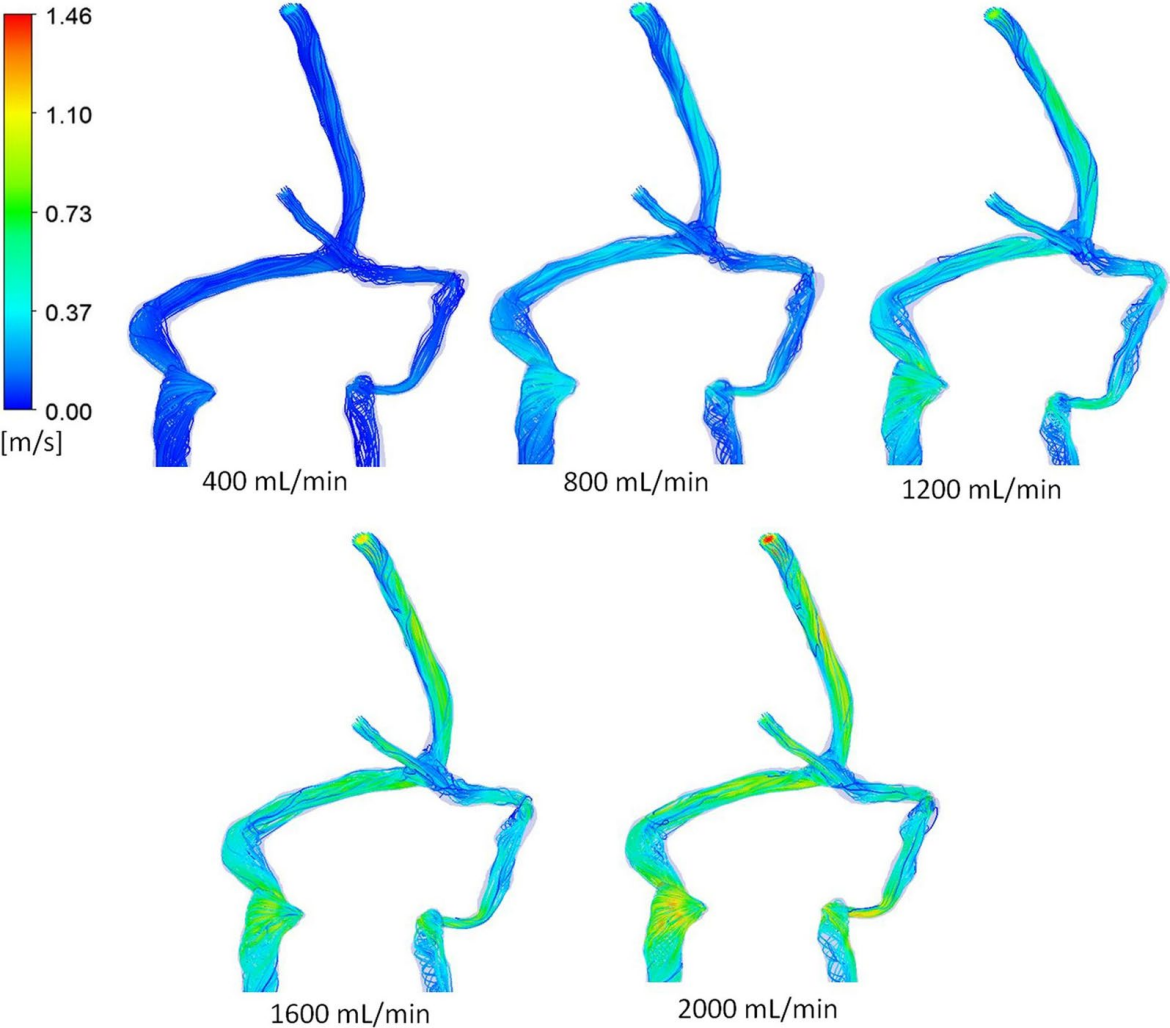
Streamline distributions throughout the cerebral venous system of patient 1 for various flow rates. The flow velocities are colour coded in m/s as per the scale. Note vortex flow increases with flow rate

**Fig. 4:**
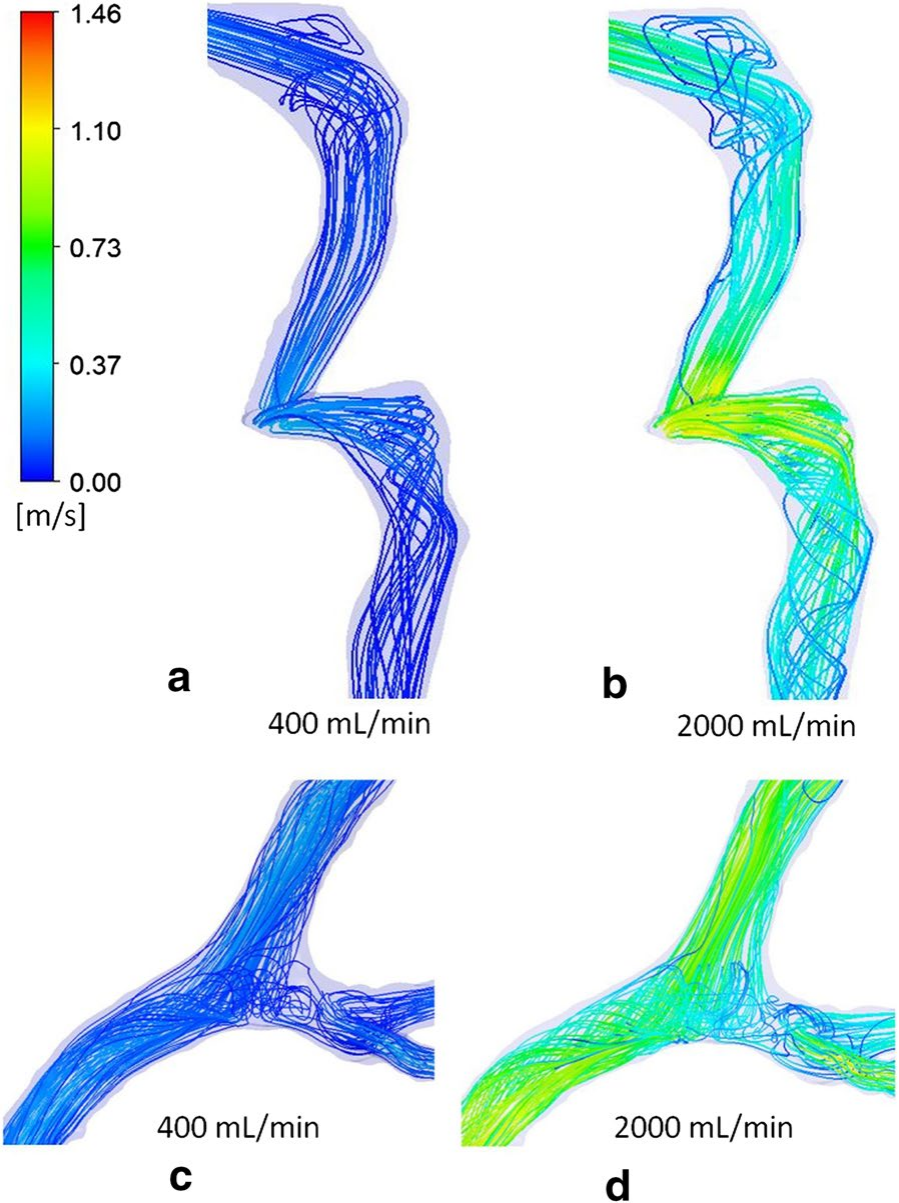
Magnified views of patient 1 with left sigmoid to internal jugular vein junction for 400 mL/min (**a**) and 2000 mL/min Flow Rates (**b**) and torcula sections for 400 mL/min (**c**) and 2000 mL/min flow rates (**d**). Note vortex flow increases with flow rate

The relationship between pressure drop across the cerebral venous system and the flow rate through the sinuses for the five patient geometries is shown in Fig. 1. No correlations between the quadratic trend lines and data points were below 0.99. The average of the 5 graphs of sinus pressure drop and flow rate was found to exceed the IIH inducing pressure threshold at approximately 1500 mL/min. The pressure related results of the CFD simulations is shown in Fig. 2, which presents the pressure distributions of patient 1, for flow rates ranging from 400 mL/min to 2000 mL/min. The quadratic relationship shown in Fig. 1 corresponds to the pressure responses shown in this diagram. The velocity streamline results of the CFD simulations are shown in Figs. 3 and 4. Figure 3 shows the blood flow streamlines of patient 1, for flow rates ranging from 400 mL/min to 2000 mL/min, relating to the pressure distributions of Fig. 2. Figure 4 highlights the increase in vortical flow of patient 1 for a flow rate of 2000 mL/min compared to 400 mL/min.

The diameter of the sinuses is summarized in Table 1. The largest standard deviation was observed to occur in the distal sigmoid sinus, with minimum observed in the superior sagittal sinus. The average curl measured at a cross-section 1.5 cm below the centre of the sigmoid sinus outlet to the jugular bulb is detailed in Table 2. The correlation between the average curl and pressure drop across the cerebral venous system was determined to be above 0.97 in all patients.

**Table 1:** Effective and hydraulic sinus diameters

Patient	SSS ${\varvec{D}}_{\varvec{h}}$ (mm)	TS ${\varvec{D}}_{\varvec{e}}$ (mm)	PSS ${\varvec{D}}_{\varvec{e}}$ (mm)	DSS ${\varvec{D}}_{\varvec{e}}$ (mm)
1	5.62	6.55	8.30	6.37
2	5.29	4.79	7.84	8.64
3	4.99	5.43	6.34	7.53
4	5.41	7.81	8.11	12.24
5	6.13	6.84	8.28	6.28
Average	5.49	6.28	7.77	8.21
SD	0.43	1.19	0.82	2.45

*DSS D*_e_ Distal Sigmoid Sinus Effective Diameter,* mm* Millimeters,* PSS D*_e_ Proximal Sigmoid Sinus Effective Diameter,* SD* Standard Deviation,* SSS D*_h_ Superior Sagittal Sinus Hydraulic Diameter,* TS D*_e_ Transverse Sinus Effective Diameter

**Table 2:** Average curl at the jugular bulb for varying flow rates

Flow Rate (mL/min)	Average Curl (${\varvec{s}}^{-1}$)				
	Patient 1	Patient 2	Patient 3	Patient 4	Patient 5
400	26.41	30.66	24.07	22.96	38.08
800	78.82	82.35	64.30	54.58	92.98
1200	159.18	115.56	106.04	91.46	154.13
1600	245.55	152.81	151.00	128.62	214.52
2000	337.99	192.79	204.05	162.55	269.55

mL/min: milliliters per minute; s^− 1^: per second

## Discussion

### **Pressure drop and flow rate relationship** 

CFD uses numerical analysis to solve fluid flows in silico. This enables CFD to be used to solve fluidic problems, whereby the original system cannot be appropriately investigated. In this study, CFD overcomes the invasive and ethically unjustifiable methods which would be required to analyse the cerebral venous system in vivo. Although manometry methods have been used to directly measure sinus pressures, the measurement of blood flow velocity and the features of this flow cannot be achieved using this technique. Thus, CFD was applied in this study to enable a more extensive understanding of this system, specifically the flow features and relationship between venous pressure and flow rate. Ten blood flows ranging from 200 mL/min to 2000 mL/min were employed, to encompass the range from a very low physiological rate to the high flow rates reported in patients with cerebral hyperemia [10]. This allowed the relationship between venous pressure and flow rate to be established, including physiologically normal and hyperemic blood flows.

In Fig. 1 the 10 data points were taken for each patient, ranging from 200 mL to 2000 mL. The horizontal red line indicates the pressure drop which must be exceeded for IIH to be induced [10]. The black line indicates the current linear model of Fall et al., which shows that regardless of flow rate, a pressure drop large enough to cause IIH would never be reached [12]. It is evident from Fig. 1 that the relationship between pressure drop and flow rate is shown to be quadratic in nature. The strong correlations of 0.99 or above between the data points and each trendline for all five patients, emphasize this trend. This differs from the previous assertion of a linear relationship in the literature and demonstrates that vascular resistance is not constant and increases with flow rate [18]. To define the reasons for this observed relationship, an investigation into the flow features was performed to correlate how they corresponded to the pressure distributions found.

The Darcy-Weisbach equation is used to quantify the losses due to fluid friction in a vessel [19]. This loss is proportional to the square of the velocity, thus predicting the quadratic relation observed. It is noted that the losses associated with the curvature of the vessels are not considered by this equation. The loss associated with a bend is, however, also proportional to the velocity squared [20]. Both equations predict the quadratic relationship which was observed in this study. Although both the losses associated with friction and curvature of the sinuses affect the total pressure drop across the system, it is evident that the vortical flow (induced by the venous curvature) is the more significant. This is because the vorticity of the flow correlated very well with the pressure drop and it would be expected that the pressure gradient along the sinuses would have been more uniform if the friction losses were more significant. Rather than the pressure drops being mostly at the tighter bends.

From Fig. 3, it is possible to observe the areas where the highest velocity increases occur and the fluid features associated with these regions. An example is at the jugular bulb to internal jugular vein sections. This is particularly apparent in the left side of patient 1 and can be observed in the five differing flow rates of Fig. 3. A magnified diagram of this section is given in Fig. 4. It is evident that at a low flow rate of 400 mL/min, a minimal amount of vortical flow is observed at the jugular bulb section. The spiralling then proceeds to dissipate at the outlet of the model. The prevalence of vortical flow is observed to increase with flow rate and can be directly compared at the 2000 mL/min blood flow. For this increased flow rate, the vorticity of the flow is more intense and continues throughout the section of internal jugular vein as modelled. It is noted that the generation of vortical flow at this segment of the venous outflow has been previously reported [17, 21, 22]. The increase in velocity of the fluid (due to the increased flow rate), causes a considerable increase in the vortical flow throughout this section, which has not been previously shown. This is a significant finding, as vortical flow is associated with higher energy losses, pertaining to proportionately larger pressure drops at higher flow rates [23]. This drop in pressure is highlighted by the substantial decrease in pressure across this section, as shown in Fig. 2. As such, it is evident that the increase in pressure drop across the cerebral venous system for larger flow rates (and fluid velocity), is related to the increased prevalence of vortical flow at the jugular bulb and internal jugular vein section.

The observation of increased vortical flow is not just isolated to the jugular bulb and internal jugular vein section. Figure 4 highlights a magnified comparison of the flow streamlines at the torcula section of the cerebral venous system. An increase in vortical flow is apparent for the higher flow rate of 2000 mL/min. This is associated with more turbulent mixing at the torcula, due to the increased fluid velocity, compared with the 400 mL/min flow rate. The vortices generated in this mixing of fluid continue into the transverse sinus in the 2000 mL/min flow rate model. In the 400 mL/min diagram, however, the vortical flow occurrence during mixing diminishes throughout the transverse sinus. Similar to the findings of the jugular bulb to internal jugular vein section, this is associated with a higher pressure drop of this segment, evident in Fig. 2. These findings explain why the pressure drop across the system increases at a higher rate for elevated flow rates through the cerebral venous system, as vascular resistance increases with an increased fluid velocity, caused by energy losses induced by the vortical flow.

As the increase in vortical flow for higher flow rates is prevalent throughout sections of the cerebral venous system, the vorticity of flow at a specific section was quantified, to establish a relationship between vorticity and sinus pressure drop. Curl, in the fluid mechanics field, corresponds to the rotation of the fluid velocity field and is a measure of vorticity [24]. The formula which defines the curl of a velocity vector field is denoted by Eq. 3.

3 $$\nabla \cdot \varvec{V}=\left|\begin{array}{ccc}i& j& k\\ \frac{\partial }{\partial x}& \frac{\partial }{\partial y}& \frac{\partial }{\partial z} \\ u& v& w\end{array}\right|=\left| \begin{array}{cc}\frac{\partial }{\partial x}& \frac{\partial }{\partial y}\\ u& v\end{array} \right| \left(in\; two \;dimensions\right)$$

where $\nabla \cdot V$ is the curl of the velocity vector field (${s}^{-1}$), $\frac{\partial }{\partial x}$ is the partial derivative with respect to x (${m}^{-1}$),

$\frac{\partial }{\partial y}$ is the partial derivative with respect to y (${m}^{-1}$), u is the velocity in the x direction (m/s) and v is the velocity in the y direction (m/s).

The fluid curl at a cross-section 1.5 cm below the centre of the sigmoid sinus outlet to the jugular bulb was measured. A correlation between the two parameters was performed and resulted in all correlations being above 0.97, indicating very strong correspondence. As such, it was shown through quantitative and qualitative means that the vortical flow prevalent in the cerebral venous system, directly corresponds to the quadratic relationship between flow rate and pressure drop observed.

### **Potential diagnostic outcomes** 

In Fig. 1, the red line indicates the absolute pressure drop that, if exceeded, would result in IIH being induced. The conventional clinical belief is that IIH could not be caused by purely elevated blood flow, suggesting high blood flow is not a significant factor in the diagnosis of IIH [10]. This is despite higher blood flow being shown to correlate in the literature with this disease and in general, no action is currently being taken to alter the criteria for clinical diagnosis [25]. The determination of a quadratic relationship between pressure drop and blood flow rate, however, implies that at flow rates higher than the population average, the venous pressure is much larger than previously expected.

While it had been suggested that the superior sagittal sinus pressure was constant over the entire lifetime of an individual [5, 26, 27], this belief has since been disproven, as it was shown that the pressure of the sagittal sinus was found to reduce with age [18]. This is due to the flow through the sagittal sinus decreasing from a mean of 620 ml/min in young patients (mean of 10 years old), to a level of 360 ml/min in middle age (mean of 44 years old) [18]. Subsequently, it was suggested that the outflow resistance was constant, and the pressure reduced with age due to changes in blood flow rate, in a linear relationship. As discussed in the background, the venous outflow resistance has been previously determined [12]. By applying this linear model, however, it is noted that the pressure drop does not follow the three known points from the literature [10]. This discrepancy is likely due to inaccuracies induced in the derivation of the resistance. The application of Poiseuille’s equation requires laminar flow through vessels which are straight with no curvature [28]. By referring to Fig. 2, it is observed that the individual sinuses do exhibit curvature, which is substantial in sections and from Fig. 3, it is noted that vortical flow is induced, especially at higher flow rates. As such, it is likely that these factors have impacted the accuracy of the method used by Fall et al. [11]. This study overcomes these issues and suggests that by incorporating the increase in blood flow vorticity for higher flows, the pressure drop can be more accurately modelled, hence yielding the quadratic relationship which agrees with the known values of the literature [10].

In four of the five patients, the pressure exceeded that which would be required to induce IIH, at blood flows which have been shown to occur in children with cerebral hyperemia [10]. In this study, 13 out of 42 or 31 % of children investigated for possible IIH had a blood flow above 1500 mL/min. This indicates that 13 patients developed cerebral blood flows which would indicate a sufficient SSS to internal jugular vein (IJV) pressure drop to develop IIH. As such, this study has shown that high blood flow is a potentially significant factor in the diagnosis of IIH. Therefore, it should be considered if a patient presents with symptoms which correspond to a potential IIH diagnosis, but with no venous stenoses or obesity. Currently, there is evidence to suggest that patients with high blood flow are not being diagnosed with IIH due to the absence of stenoses in the sinuses, because of the disregard of these findings in the literature [25, 29].

## **Limitations** 

The limitations of this study were primarily associated with the assumptions used in the CFD model. The use of a constant density indicates that the blood flow is assumed incompressible, with constant viscosity indicating a Newtonian flow assumption. The values used have been measured and are an accurate representation of the average human blood properties [30]. Incompressible flow is a simplification which is acceptable because the changes in pressure, which would cause density gradients in the flow, are minimal. As the fluid velocities are significantly below the speed of sound, incompressible flow is an accurate representation of the system [31]. A comparison of a Newtonian and Non-Newtonian fluid through the human aorta found that the flow patterns were comparable, with an approximate error of 4 % in wall shear stress values [32]. As such, incompressible, Newtonian flow was implemented in this study.

Rigid walls were another assumption used in this study, as intracranial veins run through the meninges, a quasi-incompressible biological tissue composed mostly of water, and are not susceptible to experience large deformations at normal transmural pressures [33]. Further, in a study of 20 normal and IIH patients, it was found that the cross-sectional area of the sagittal sinus was almost identical (< 1 % difference) [34]. This indicated that the transmural pressure was likely to remain normal despite the increase in venous pressure associated with IIH, as the ICP increases in tandem to the venous pressure. The rigid wall assumption was employed to reduce the complexity of the model (and hence solving times); however, it was not incorporated under the belief of the sinus walls being perfectly rigid. Rather that the transmural pressure is expected to remain constant and therefore the sinus wall will not altering in position (hence constant cross-sectional areas), which is expected in normal patient such as those modelled in this study. As such, it has been determined that the venous walls are to be assumed rigid throughout this study.

An additional assumption was the use of steady flow rather than pulsatile. This has been previously found to be an appropriate simplification for CFD simulations investigating IIH in the cerebral venous system [35]. Furthermore, it has been shown that the pulsatility index of blood flow in the cerebral venous system is significantly lower than in the arterial system, with a reduction of 43 % observed in the control group in the sagittal sinus compared to the arterial [25]. It was also found that the pulsatility index decreased further with increased blood flow through the system. This is an important finding for this study, as blood flows which are significantly above normal levels were simulated. As such, it is expected that the pulsatility of the blood flow throughout this system will be minimal.

Another limitation is that only adult CT data was available and not data from children. CT data was used because of the inherent high resolution. The study was not performed using pediatric CT venograms as there were none available for use as the radiation dose associated with CT venograms is considered too high for use in children at the hospital from which the images were obtained and MR imaging is preferred for children (however this resolution is lower and was hence not used in this study). However, the average effective diameters of the venous segments of the CT patients used were not significantly different to the average effective diameters of the children with hyperemic IIH from the previous paper [10], indicating the geometries were comparable. Following 4 years of age, the sinuses had largely attained full size with no significant difference in the second and third controls groups for any of the sinuses [10]. The CT data was only obtained with the patients in the supine orientation, and no upright imaging was acquired. This would not likely affect the intracranial venous geometry modelled, as it has been previously shown that no significant changes in the sizes of the sinuses were observed in supine and upright CTs [36].

The validation process indicated that the models used were a valid representation of the population average,  irrespective of the assumptions employed. Despite this, it is important to appreciate that there is accuracy lost in the use of simplifications. Another limitation of this study was that the sample size was relatively small, with five patients investigated. The high correlations between the trendlines and data points of each individual patient, however, strongly suggest that the quadratic relationship found is an accurate representation of the flow and pressure response. Furthermore, the successful validation with the known points in the literature aid the accuracy of the study. Irrespective of this, the expansion of this analysis to include additional patients would strengthen the reliability of the results found.

A significant limitation was the removal of small cortical veins from the patient geometries. Despite only supplying a relatively small volume of blood to the system, their removal reduces the accuracy of the flow features, whereby additional mixing would be observed throughout the cerebral venous system. This would, however, likely increase the pressure drops observed by a small amount, as the superficial cortical veins enter the sagittal sinus obliquely and the direction of flow is usually opposite to that within the sinus [37]. As such, the entering fluid would disrupt the blood flow of the sagittal sinus, as the direction is first required to be reversed to that of the main flow, thus increasing the venous pressure [37]. The veins were removed as they vary significantly between patients and the proportion of blood flow which is supplied by them is not known, making population averaged values not feasible to determine [37]. There was no accurate method of including these veins and hence they were removed. This has additional implications for the model, as the known total combined blood flow at the inlets and outlets of the modelled system differs by 23 %. This proportion is the total flow supplied by the combination of the additional cortical veins [38, 39]. To ensure the continuity of the model, which is a requirement of CFD, an average of the inlet and outlet flow rates was used. The validation process suggested that this was an acceptable simplification, however regardless, it is a limitation of the study. It is noted that a Starling resistor mechanism is associated with the cortical veins that bridge the sub-arachnoid space [40]. The pulse waves which can be introduced into the venous flow due to this system was not modelled. The CFD models used in this study are a simplification of the true system and therefore the absence of incorporation of the Starling resistor mechanism is a limitation of this study.

## Conclusions

CFD modelling has shown that the pressure response of the venous sinus system to cerebral blood flow is quadratic. The major determinant appears to be the degree of rotation induced in the blood flow. The increased blood flow found in children with IIH can account for the increased ICP, secondary to the increase in venous pressure that develops.

## Data Availability

All data generated or analysed during this study are either included in this published article available at reasonable request.

## Abbreviations

CBF: Cerebral blood flow
CFD: Computational fluid dynamics
CNS: Central nervous system
CVP: Central venous pressure
D_e_: Effective diameter
FR_csf_: CSF formation rate
Hd: Hydraulic diameter
ICP: Intracranial pressure
IIH: Idiopathic intracranial hypertension
IJV: Internal Jugular Vein
Kgm^3^: Kilogram meter squared
mL/min: Milliliters per minute
mm: Millimeters
mmHg: Millimeters of mercury
Pa.s: Pascal seconds
R_out_: CSF outflow resistance
s^− 1^: Per second
SSS_p_: Superior sagittal sinus pressure
TCBF: Total cerebral blood flow
